# Supplementary material for: Ly6D+Siglec-H+ precursors contribute to conventional dendritic cells via a Zbtb46+Ly6D+ intermediary stage
Source: Nat Commun. 2022 Jun 16;13:3456. doi: 10.1038/s41467-022-31054-4 (PMC9200809; doi:10.1038/s41467-022-31054-4)
Supplement: Supplementary file 1 — Supplementary Information [file 41467_2022_31054_MOESM1_ESM.pdf]

Supplementary Data and Tables

Supplementary Data 1 is provided as a separate .xlsx file.

Supplementary Data 1: Enriched KEGG pathways

Supplementary Data 2 is provided as a separate .xlsx file.

Supplementary Data 2: Enriched TF motifs

Gene	Assay Number	Transcript detected	Dye	Lot	Manufacturer
Batf3	Mm01318274_m1	NM_030060.2	FAM	610932	ThermoFisher
Cd74	Mm00658576_m1	NM_001042605.1	FAM	3847897	ThermoFisher
Cx3cr1	Mm00438354_m1	NM_009987.4	FAM	816513	ThermoFisher
HPRT	Mm.PT.39a.22214828	NM_013556	HEX	365840192	IDT
Id2	Mm00711781_m1	NM_010496.3	FAM	854994	ThermoFisher
Irf4	Mm00516431_m1	NM_013674.1	FAM	1204526	ThermoFisher
Irf8	Mm00492567_m1	AK018533.1	FAM	854037	ThermoFisher
Itgam	Mm00434455_m1	NM_001082960.1	FAM	3623266	ThermoFisher
Itgax	Mm01271280_m1	NM_021334.2	FAM	P210119	ThermoFisher
Klf4	Mm00516104_m1	NM_010637.3	FAM	3473212	ThermoFisher
Ly6d	Mm00521959_m1	NM_010742.1	FAM	P210119	ThermoFisher
Siglech	Mm00618627_m1	NM_178706.4	FAM	572659	ThermoFisher
Sfpi1	Mm00488142_m1	NM_011355.1	FAM	887886	ThermoFisher
Spib	Mm01719550_s1	NM_019866.1	FAM	735002	ThermoFisher
Stat3	Mm01219775_m1	NM_011486.4	FAM	1206368	ThermoFisher
Tcf4	Mm01262526_g1	NM_001083967.1	FAM	P100419	ThermoFisher
Zbtb46	Mm00511327_m1	NM_027656.2	FAM	P210119	ThermoFisher
Zeb2	Mm00658576_m1	NM_001289521.1	FAM	P210119	ThermoFisher

Supplementary Table 1: qPCR Taqman™ Probes used in this study

Target	Fluorophore	Clone	Dilution	Manufacturer
B220	BV605	RA3-6B2	1:200	BioLegend
B220	FITC	RA2-6B2	1:200	eBioscience
B220	BV605	RA3-6B2	1:200	BD
BST-2	APC	eBio129c	1:200	eBioscience
CCR4	PE	2G12	1:200	BioLegend
CCR5	BUV737	2D7/CCR5	1:100	BD
CCR5	PE	HM-CCR5	1:200	BioLegend
CCR9	PE	eBioCW-1.2	1:200	eBioscience
CCR9	eF450	CW-1.2	1:100	eBioscience
CD115	PE-Cy7	AFS98	1:200	BioLegend
CD115	BUV737	AFS98	1:200	BD
CD115	APC	AFS98	1:200	BioLegend
CD117	BV510	ACK2	1:200	BioLegend
CD117	PE	2B8	1:200	BioLegend

CD11b	APC	M1/70	1:200	BioLegend
CD11b	PerCP-Cy5,5	M1/70	1:200	BioLegend
CD11b	BV570	M1/70	1:200	BioLegend
CD11c	PE-Cy7	N418	1:200	BioLegend
CD11c	PE-Cy7	N418	1:200	eBioscience
CD11c	APC-Cy7	HL3	1:200	BD
CD11c	BUV395	HL3	1:200	BD
CD135/Flt3	APC	A2F10	1:100	BioLegend
CD135/Flt3	BV421	A2F10	1:100	BioLegend
CD14	PE	Sa2-8	1:200	BioLegend
CD16/32	PerCP-Cy5,5	93	1:200	BioLegend
CD16/32	BUV496	93	1:200	BD
CD19	APC-Cy7	6D5	1:200	BioLegend
CD19	FITC	1D3	1:200	BD
CD2	BUV615	RM2-5	1:100	BD
CD24	APC	M1/69	1:200	BioLegend
CD24	BV605	M1/69	1:200	BioLegend
CD3	APC-Cy7	145-2C11	1:200	BioLegend
CD3	FITC	145-2C11	1:200	BD
CD4	APC	RM4-5	1:200	BioLegend
CD4	BUV661	SK3	1:200	BD
CD45.1	PE-Cy7	A20	1:200	eBioscience
CD45.1	AF700	A20	1:200	BioLegend
CD45.2	APC	104	1:200	BioLegend
CD45.2	Alexa Flour 700	104	1:200	BioLegend
CD64	APC	X54-5/7.1	1:200	BioLegend
CD81	Alexa 350	431301	1:200	Novus Bio
CD86	BV650	GL-1	1:200	BioLegend
CD8a	PerCP	53-6.7	1:200	eBioscience
CD8a	PE	53-6.7	1:200	BD
CD9	APC	eBioKMC8	1:200	eBioscience
CX3CR1	BV711	SA011F11	1:200	BioLegend
CX3CR1	PE	SA011F11	1:200	BioLegend
CXCR3	PerCP	CXCR3-173	1:100	eBioscience
CXCR4	APC	2B11	1:200	eBioscience
CXCR5	APC	L138D7	1:200	BioLegend
F480	BUV805	T45-2342	1:400	BD
IL7R/CD127	PerCP-Cy5.5	A7R34	1:200	BioLegend
IL7R/CD127	PE-Cy7	A7R34	1:200	BioLegend
Ly49Q	FITC	2E6	1:200	MBL
Ly6C	PE-Dazzle594	HK1.4	1:400	BioLegend
Ly6C	AF700	HK1.4	1:200	BioLegend
Ly6D	PE	49-H4	1:800	BioLegend

Ly6G	APC-Cy7	1A8	1:200	BioLegend
Ly6G	FITC	1A8	1:200	BioLegend
MHCII	APC-eFluor780	M5/114.15.2	1:400	eBioscience
MHCII	BV650	M5/114.15.2	1:400	BioLegend
MHCII	BV650	M5/114.15.2	1:400	eBioscience
MHCII	Spark Blue 550	M5/114.15.2	1:400	BioLegend
NK1.1	APC-Cy7	PK136	1:200	BioLegend
NK1.1	FITC	PK136	1:200	BD
Sca1	APC	D7	1:200	BioLegend
Siglec-H	BV786	440c	1:200	BD
Siglec-H	AF488	440c	1:200	BioLegend
Siglec-H	FITC	440c	1:200	BioLegend
Siglec-H	AF647	440c	1:200	BioLegend
Sirp-alpha	PerCP-Cy5.5	P84	1:200	BioLegend
Sirp-alpha	Alexa Flour 700	P84	1:200	BioLegend
Ter119	APC-Cy7	TER-119	1:200	BioLegend
Ter119	FITC	TER-119	1:200	BioLegend
XCR1	Alexa Flour 647	ZET	1:200	BioLegend

Supplementary Table 2: Antibodies used in this study

Supplementary Methods

IFN-α ELISA

For IFN-α production assays Ly6D$^+$ Zbtb46$^-$ lo-lo and lo-hi cells were sorted, cultured on EL08-1D2 stromal cells with 3% Flt3L containing SN for 3d followed by stimulation with 0,5 µM CpG-A for 24h. SNs from stimulated lo-lo, lo-hi and EL08 cells as control were analyzed for IFN-α concentration using a high-sensitivity VeriKine™-HS Mouse Interferon Alpha All Subtype ELISA Kit (pbl assay science, Cat. No: 42115-1) according to the manufacturers protocol. ELISA data were analyzed in Prism utilizing a 4-parameter logistical fit for the standard curve as proposed in the manufacturers protocol.

CDP/Ly6D$^+$ Siglec-H$^+$ LP sort

CDP and Ly6D$^+$ Siglec-H$^+$ LP were sorted from Lin$^-$ (CD3, CD19, CD105, NK1.1, Ly6G) CD16/32$^-$ CD11c$^-$ B220$^-$ Ly6C$^-$ CD135$^+$ CD117^{lo-int} BM cells of Zbtb46-eGFP$^{wt/ki}$ mice. CDP were sorted as CD115$^+$ IL7R$^-$ and Ly6D$^+$ Siglec-H$^+$ LP were sorted as CD115$^-$ IL7R$^+$ Ly6D$^+$ Siglec-H$^+$. CDP were cultured on EL08-1D2 stromal cells with 5% Flt3L containing SN for 4d. Ly6D$^+$ Siglec-H$^+$ LP were cultured without stromal cells in 96-well plates with 10% Flt3L containing SN for 4d, then analyzed by flow cytometry.

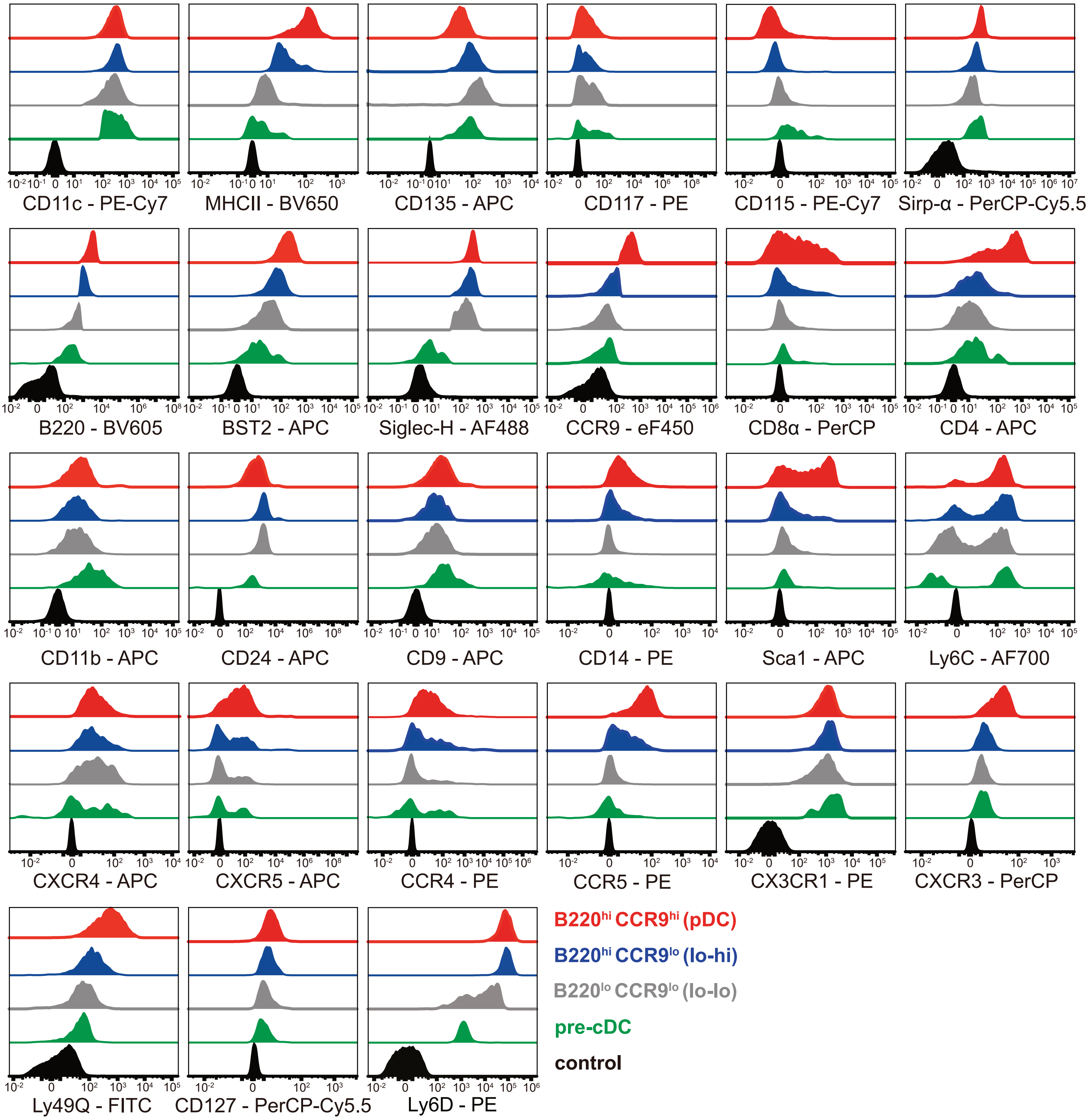

Supplementary Figure 1. Surface marker expression in pDCs and precursor subsets

Comparison of surface marker expression in pre-cDC, lo-lo, lo-hi and pDC in the BM of C57BL/6 mice. BM single cell suspensions were stained with fluorescently labeled antibodies against the indicated cell surface markers as described in the methods section without prior enrichment steps. Histograms showing fluorescence intensity of surface markers measured by flow cytometry (normalized to mode, unstained cells as control). Representative results of 3 independent experiments.

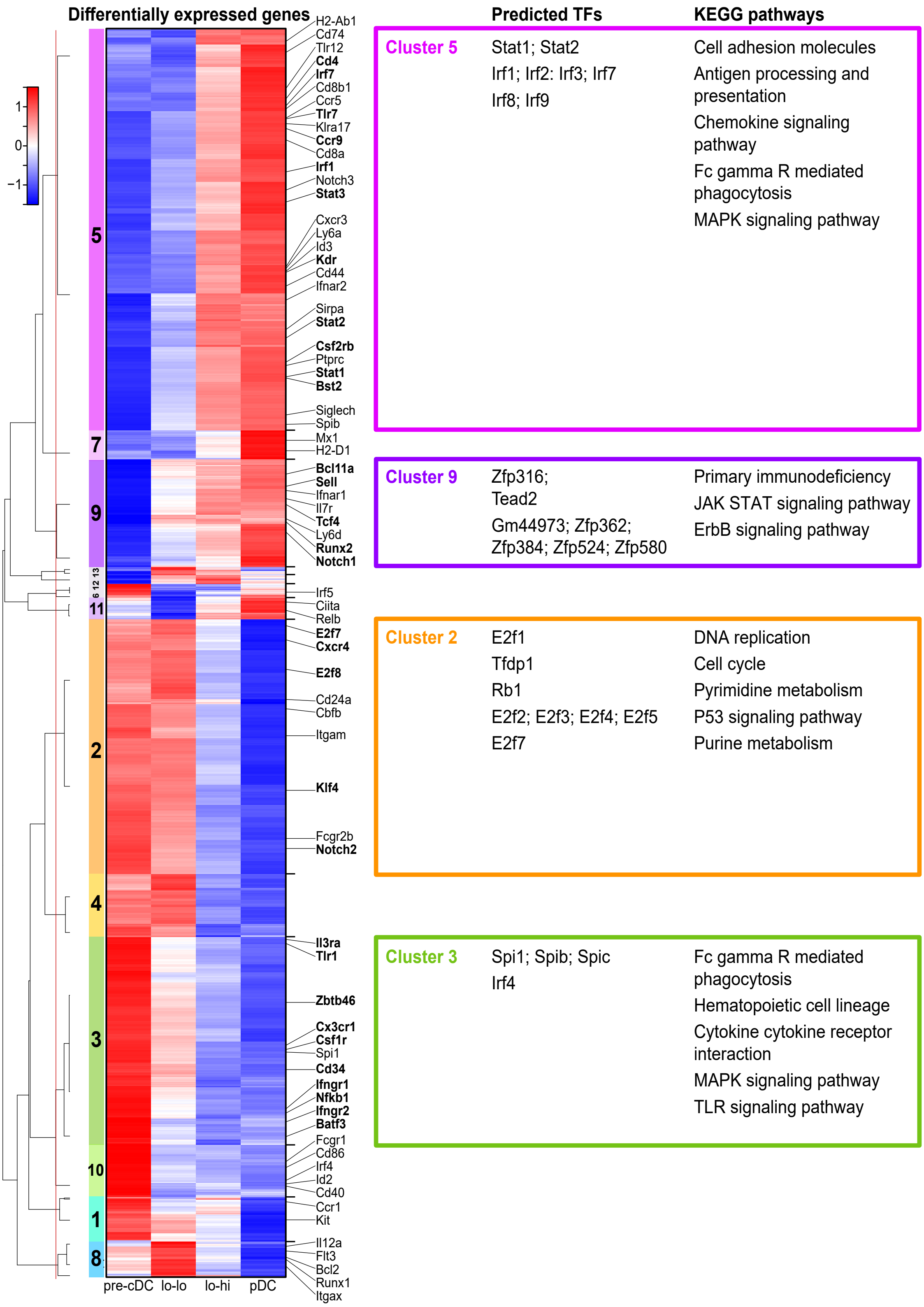

Supplementary Figure 2. Gene expression heatmap of DC precursors

pre-cDC, lo-lo, lo-hi and pDC were sorted from the BM cells of 3 individual C57BL/6 mice and processed for RNA-sequencing. Expression heatmap of DEG, hierarchically clustered by Euclidean distances between scaled mean values (normalized counts). Left panel: Heatmap of 2880 DEGs (likelihood ratio test, Benjamini-Hochberg adjusted p value < 0.01) with 13 clusters of co-regulated genes. Genes in bold letters are known targets for the transcription factors (TFs) shown on the right. Colors indicate row z-scores. Right panel: Shown are the known TFs that bind to motifs enriched in the major clusters (selected for highest enrichment scores for each cluster) and selected KEGG functional pathways enriched in the major clusters.

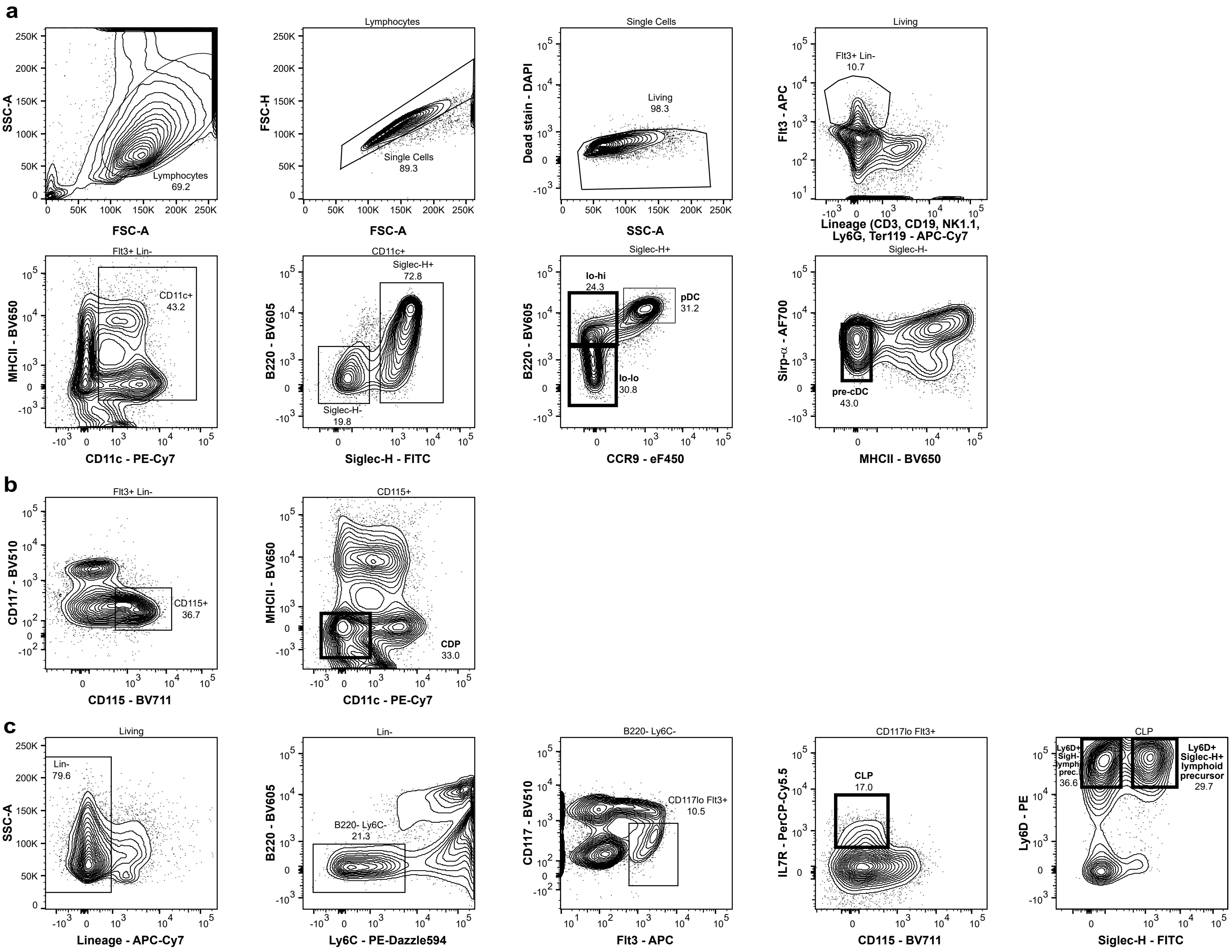

Supplementary Figure 3. Gating strategy for sort populations for scRNA-sequencing

The following cell populations were sorted from Lineage-depleted BM cells: (a) lo-lo, lo-hi, pDC and pre-cDC. (b) CDP. (c) CLP, Ly6D^{+} and Ly6D^{+} Siglec-H^{+} lymphoid progenitors. Gating strategies are depicted from left to right, followed by the next row, where applicable. Parent gates are indicated above the contour plots.

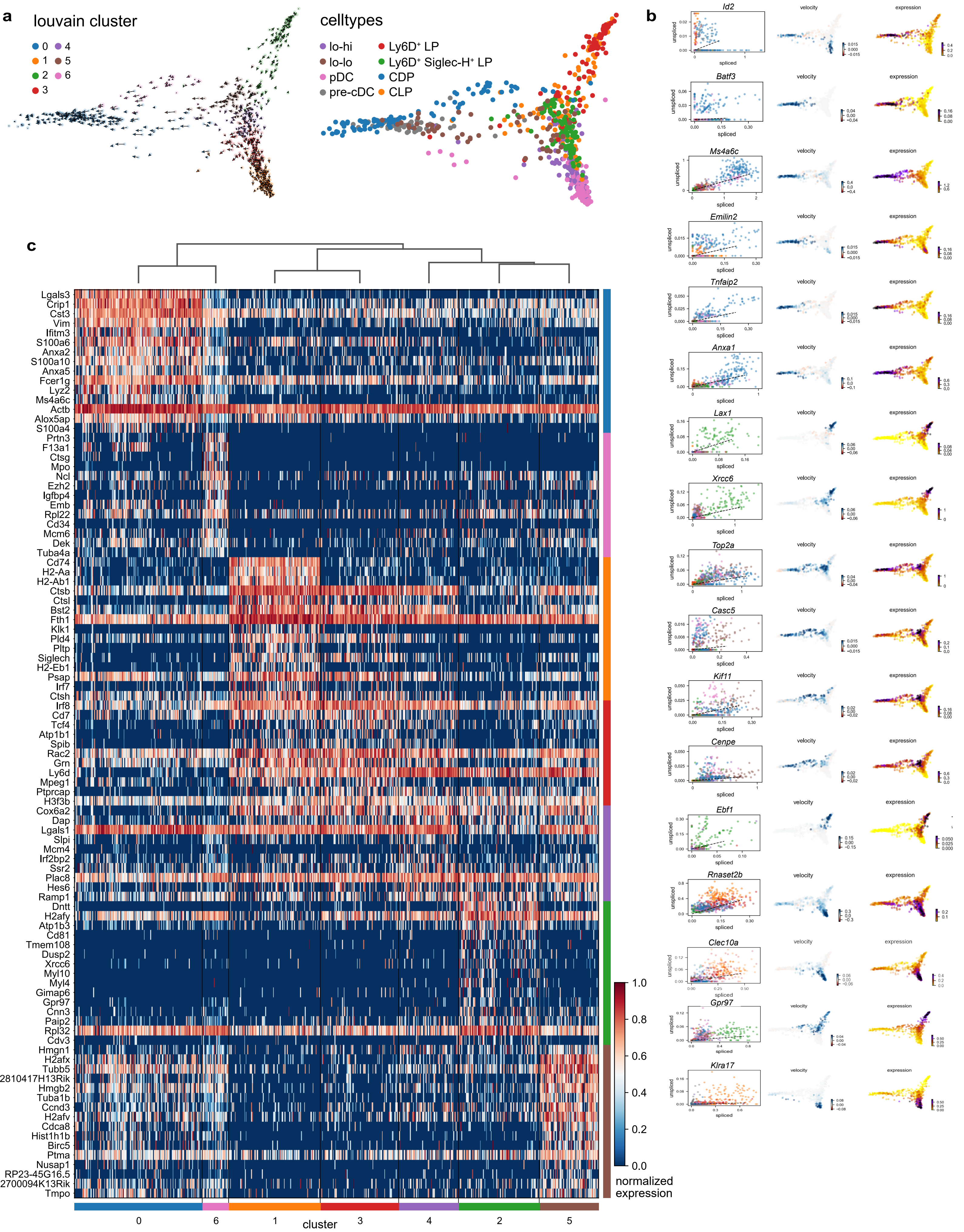

Supplementary Figure 4. Transcriptional dynamics of DC progenitor cells in murine BM (a) Clusters were identified by Louvain community detection and cells were highlighted in their respective cluster colours on the diffusion map (left) or highlighted in their respective sorted identity (right). Arrows illustrate calculated RNA velocity on a single-cell level. Longer arrows indicate higher velocity. (b) Spliced/unspliced ratios, velocity and expression of selected genes indicated by colours. (c) Heatmap of top 15 ranked velocity driving genes per cluster (indicated by corresponding color on the right Y-axis), duplicates excluded. Expression values normalized between 0 and 1.

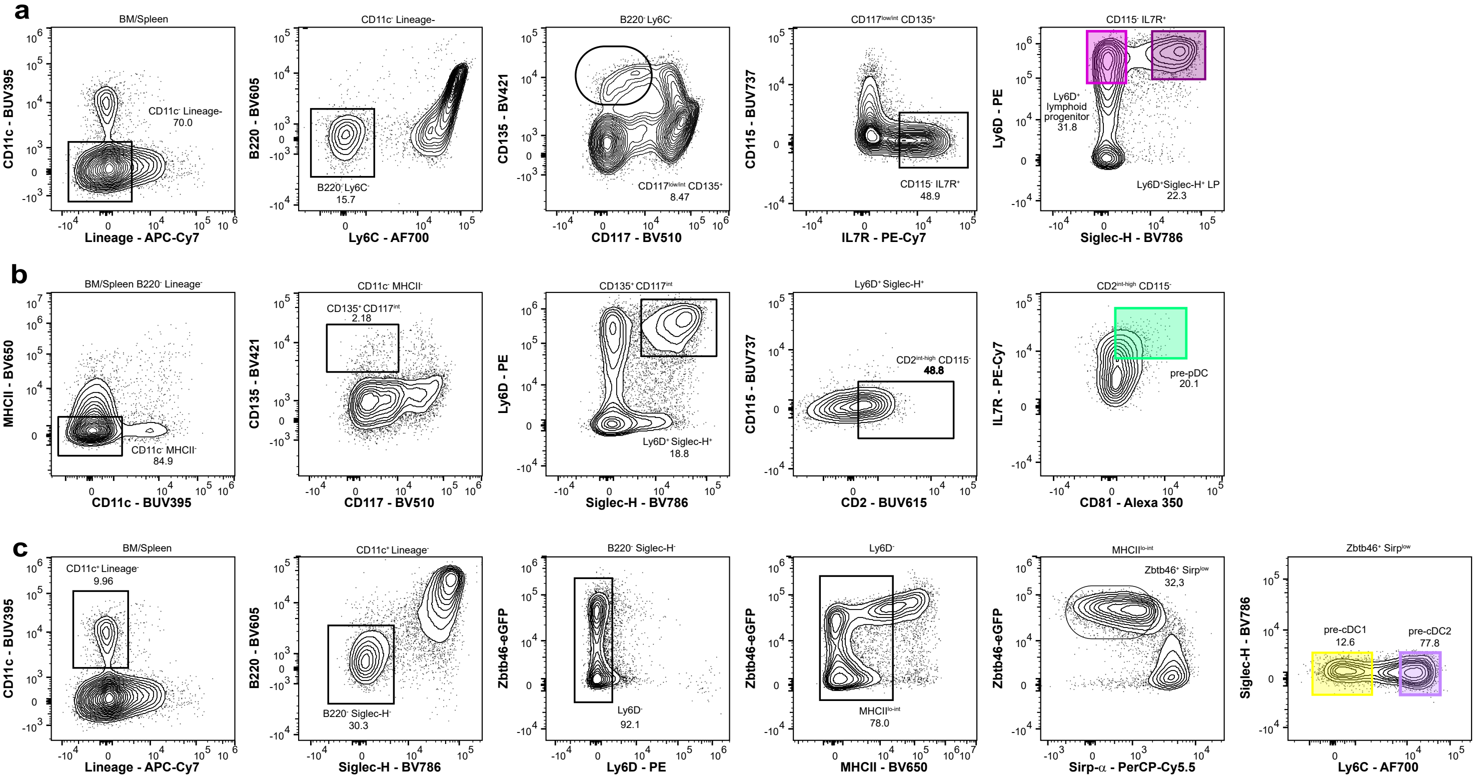

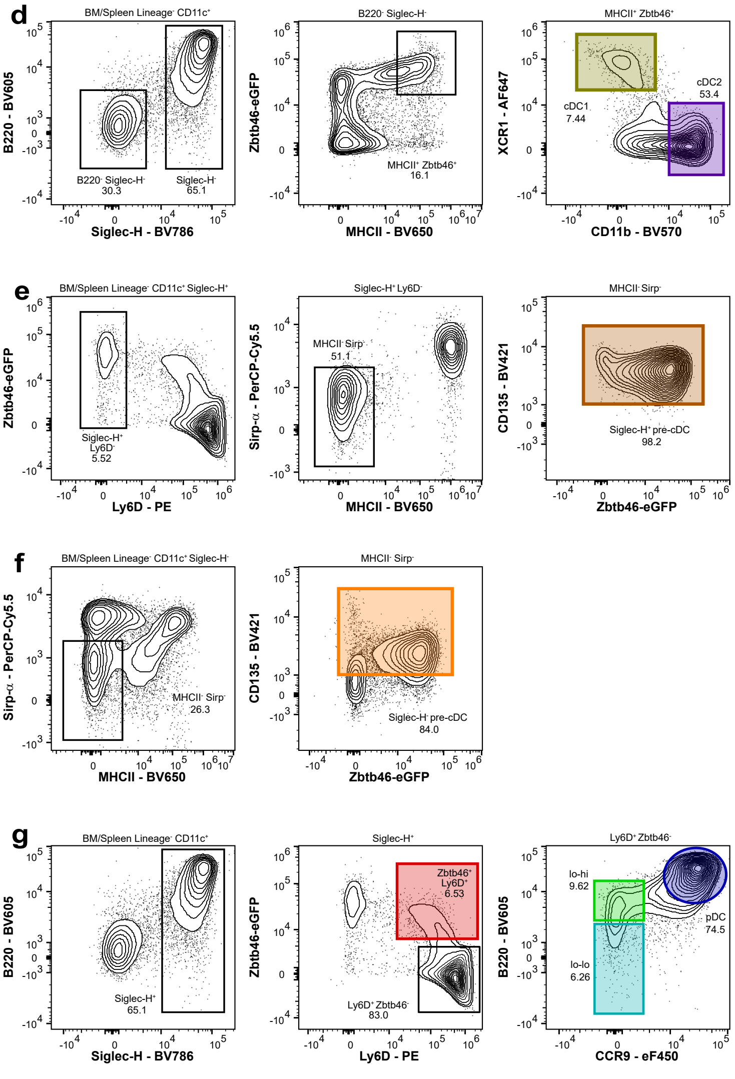

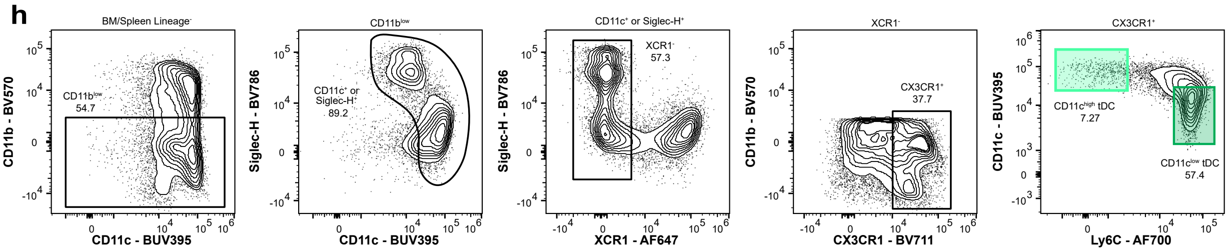

Supplementary Figure 5. Gating strategy for multi-parameter spectral flow cytometry analysis of steady-state BM and spleen

Multidimensional flow cytometric analysis with 25 fluorophores. BM and spleen cells from Zbtb46-eGFP$^{wt/ki}$ mice enriched for lineage$^-$ cells were acquired separately and concatenated for analysis. To project cell types onto the UMAP generated in Fig. 3 they were gated from Lineage$^-$ BM/Spleen as follows. Gating strategy for: (a) Ly6D$^+$ and Ly6D$^+$ Siglec-H$^+$ lymphoid precursors. (b) pre-pDC. (c) pre-cDC1 and pre-cDC2. (d) cDC1 and cDC2. (e) Siglec-H$^+$ pre-cDC. (f) Siglec-H$^-$ pre-cDC. (g) Zbtb46$^+$ Ly6D$^+$ cells and Ly6D$^+$ Zbtb46$^-$ lo-lo, lo-hi and pDC. (h) tDC. Gating strategies are depicted from left to right.

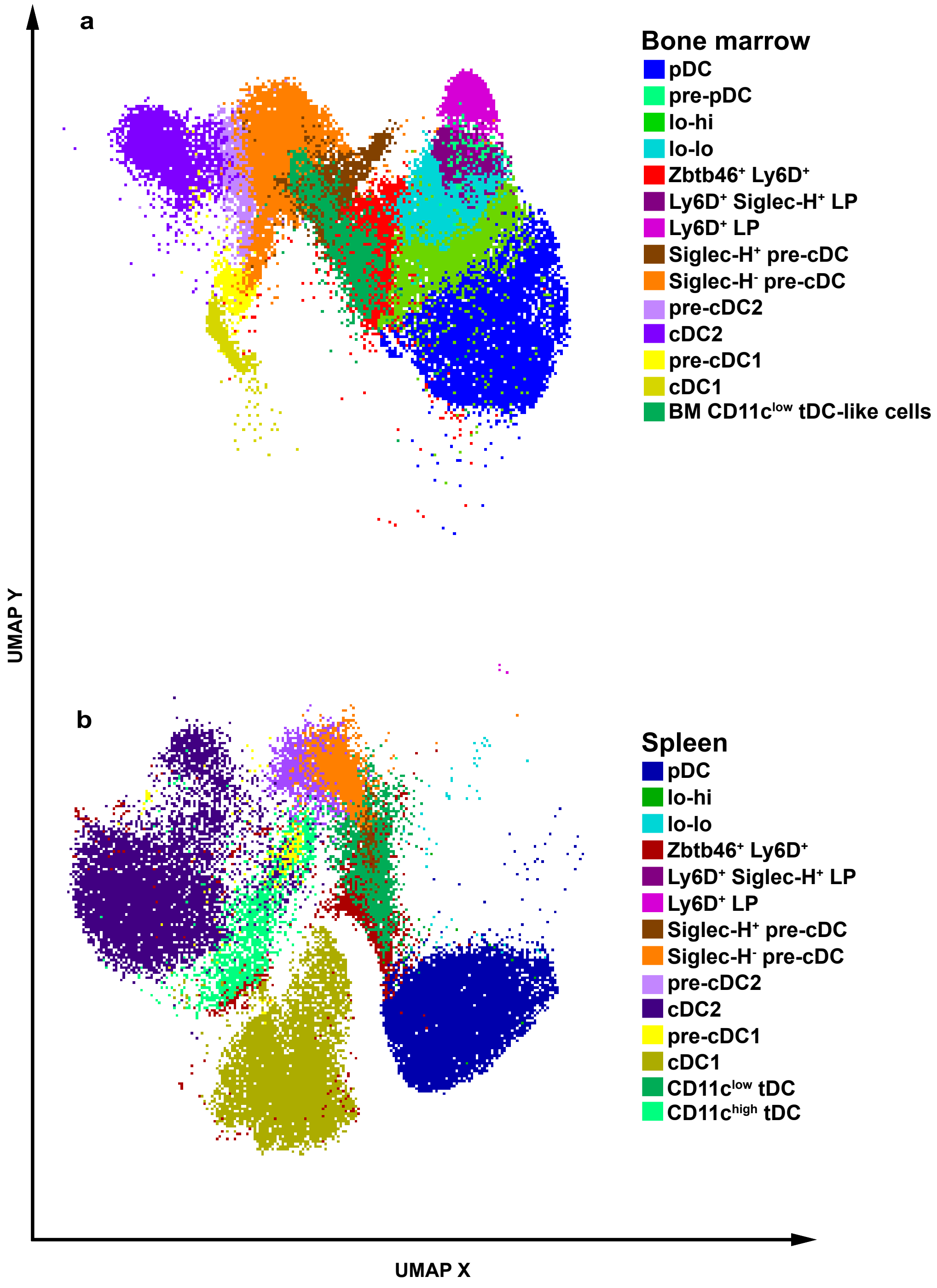

Supplementary Figure 6. Phenotypic relation of DC subsets and DC precursors in BM and spleen. Lineage-depleted BM and spleen cells of Zbtb46-eGFP$^{wt/ki}$ mice were analysed by multidimensional spectral flow cytometry (25 parameters). (a) Manually gated BM DC subsets, precursors and Zbtb46^{+} Ly6D^{+} cells projected onto the UMAP of Fig. 3c, top. (d) Splenic DC subsets, precursors and tDC (for gating see Fig. S8) projected onto the UMAP of Fig 3c, bottom.

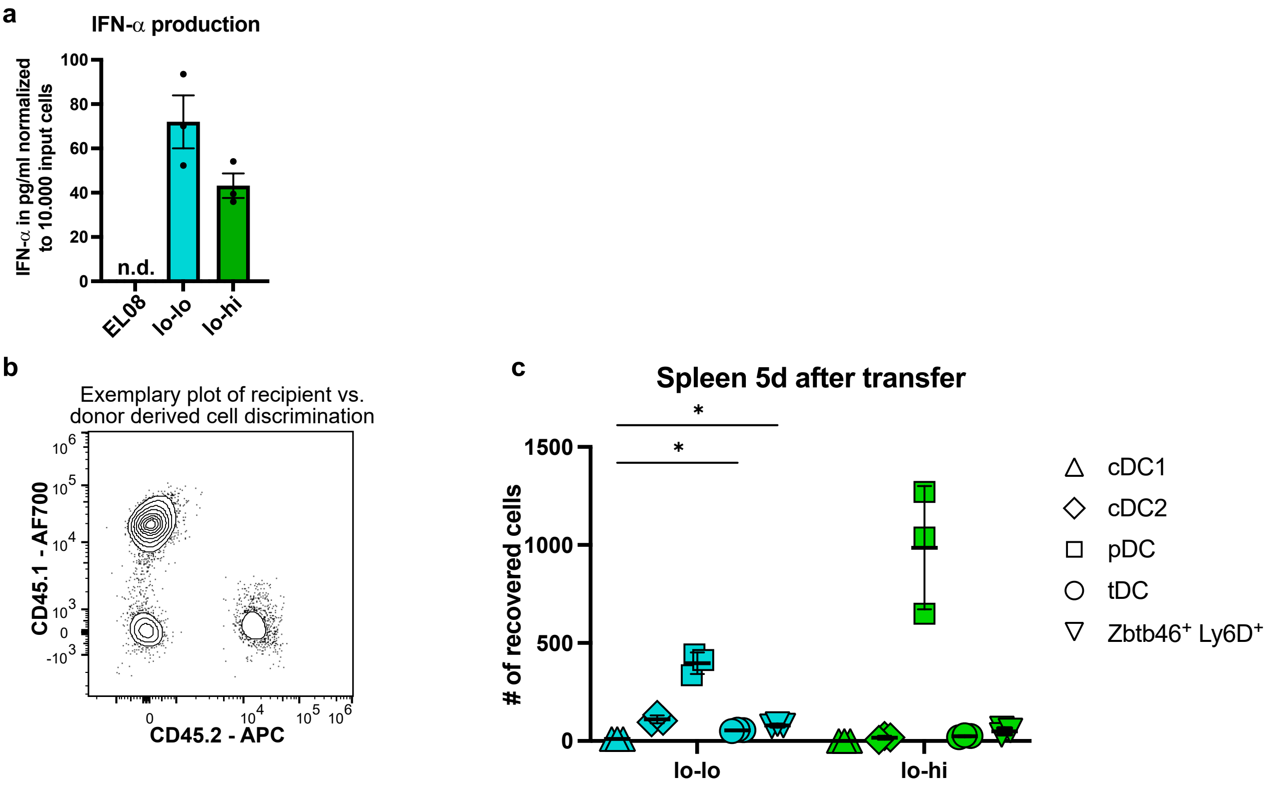

Supplementary Figure 7. Function of generated pDC and in vivo fate of pDC precursor cells

(a) $Ly6D^+$ $Zbtb46^-$ lo-lo and lo-hi cells were sorted from BM of Zbtb46-$eGFP^{wt/ki}$ mice (male, C57BL/6 background, 9-15 weeks old) , cultured for 3d on EL08-1D2 stromal cells and stimulated with 0.5 µM CpG-A for 24h. SN was collected and IFN-α concentrations were measured by ELISA. Normalised IFN production is shown as mean ± SEM (n=3, n.d. = not detectable). $Ly6D^+$ $Zbtb46^-$ lo-lo and lo-hi cells were sorted from BM of Zbtb46-$eGFP^{wt/ki}$ mice (C57BL/6 background, female, 8-12 weeks) and injected i.v. into the tails of CD45.1 congenic mice (*Ptprc*^{a}, C57BL/6 background, male, 8-10 weeks). On d5 after injection recipient mice were sacrificed, Spleens were harvested and analyzed by flow cytometry. Injected cells were identified by CD45.2 expression (b, cells enriched for donor derived cells for better resolution). (c) Recovered pDC , cDC1 , cDC2, tDC and $Zbtb46^+$ $Ly6D^+$ cell counts are shown with mean ± SEM (n=3). Counts were compared for each input population using a 2-Way ANOVA with Holm-Šídák correction for multiple testing (adjusted p-values: <.05(*).

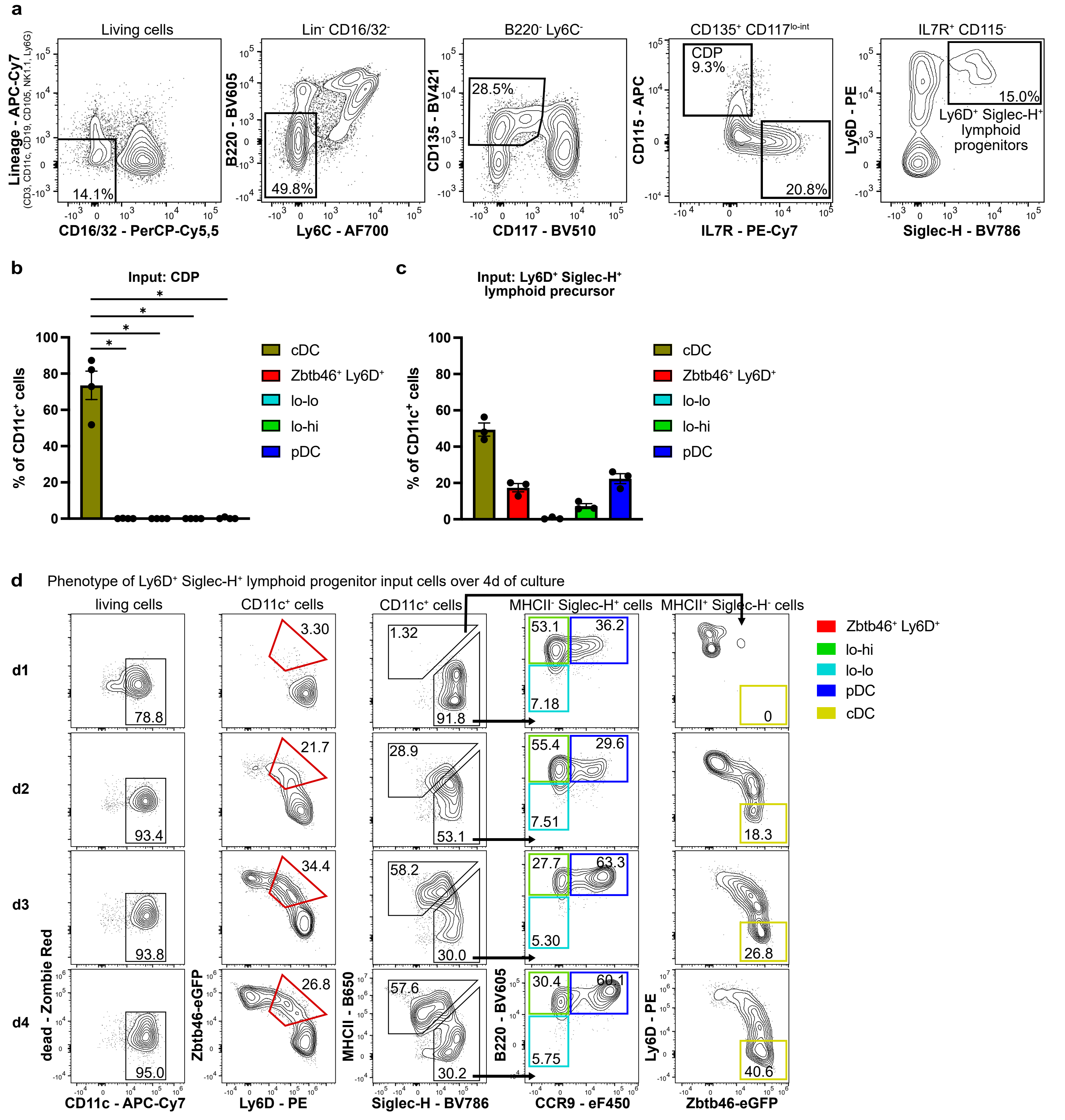

Supplementary Figure 8. Cell fate of earlier precursor cells

(a) Gating strategies for CDP and Ly6D⁺ Siglec-H⁺ lymphoid progenitor sorts. (b) Cell fate of CD115⁺ IL7R⁻ CDP cultured for 4d on EL08-1D2 stromal cells with Flt3L shown as percentage of total CD11c⁺ cells as mean ± SEM (n=4). Cell type output was compared using paired, two-sided t-tests with Holm-Šídák correction for multiple testing (adjusted p-values: <.05(*). (c) Cell fate of Ly6D⁺ Siglec-H⁺ lymphoid precursors cultured with 10% Flt3L containing SN without stromal cells for 4d, shown as percentage of total CD11c⁺ cells as mean ± SEM (n=3). (d) Phenotype kinetics of cells generated by Ly6D⁺ Siglec-H⁺ lymhoid precursor cells as input, cultured with 10% Flt3L over the course of 4d.

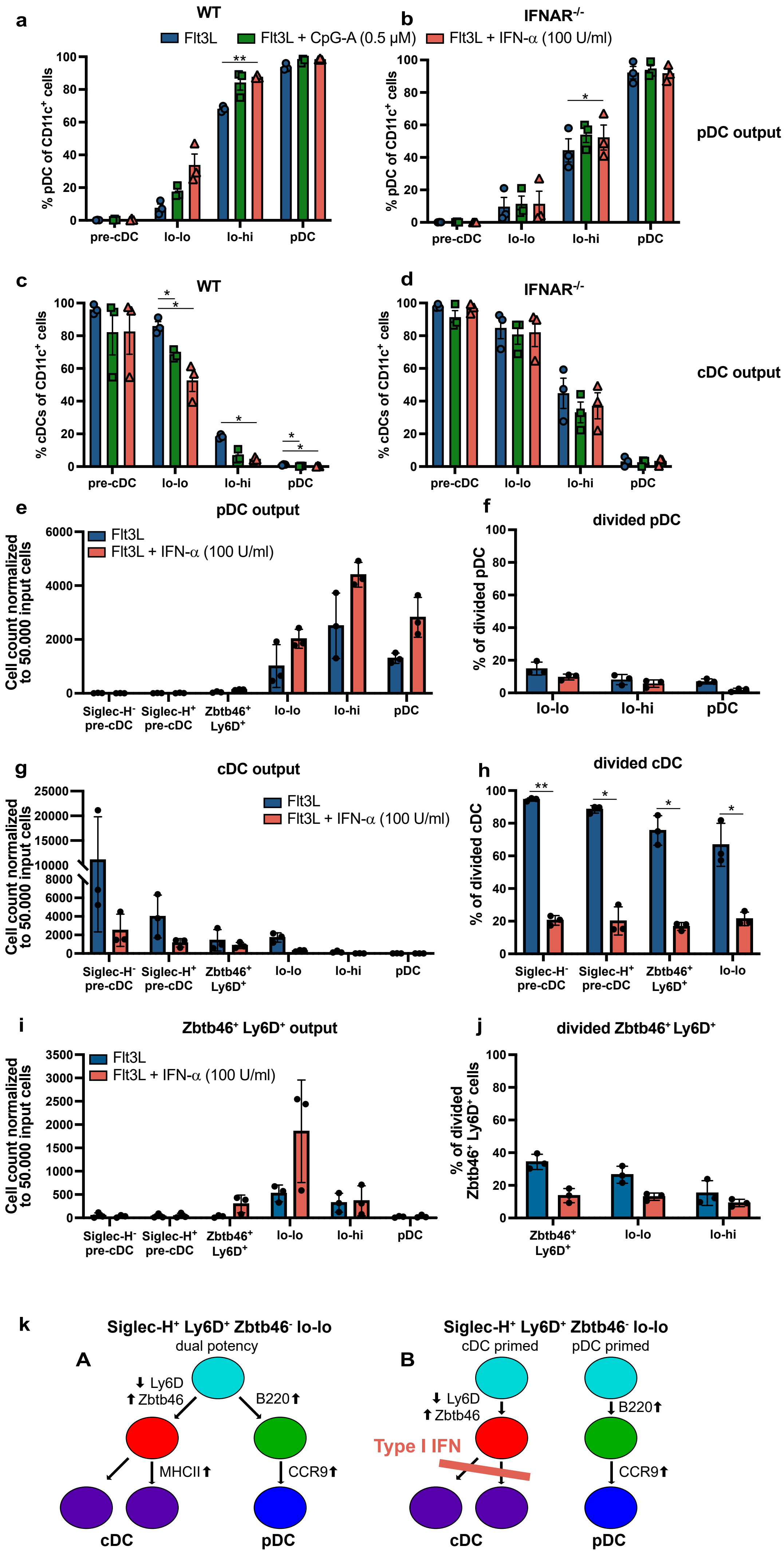

Supplementary Figure 9. Effects of stimulation on DC precursor cell output and proliferation

Siglec-H^{-} pre-cDC and lo-lo, lo-hi and pDC were sorted from Lineage-depleted BM cells from WT mice (a, c) or IFNAR$^{-/-}$ mice (b, d) and cultured on EL08 cells with Flt3L alone or with CpG or Type I IFN. Percentages of pDCs (a, b) and cDCs (c, d) are shown as mean ± SEM (n=3). (e, g, i) Siglec-H^{-} pre-cDC, Siglec-H^{+} pre-cDC, Siglec-H^{+} Zbtb46^{+} Ly6D^{+} cells and lo-lo, lo-hi and pDC (gated Siglec-H^{+} Zbtb46^{-} Ly6D^{+}) were sorted from Lineage-depleted pooled BM cells of Zbtb46-eGFP$^{wt/ki}$ mice and cultured for 3 days with Flt3L on EL08-1D2 stromal cells with or w/o addition of IFN I (n=3). The phenotype was analyzed by flow cytometry. Cell counts (normalized to 50,000 input cells) are shown for pDC (e), cDC (g), and Zbtb46^{+} Ly6D^{+} cells (i) as mean ± SEM (n=3). Cells were labelled with CellTrace Blue proliferation dye before the sort. Divided and undivided cells were gated for pDC (f), cDC (h) and Zbtb46^{+} Ly6D^{+} cells in Flt3L and Flt3L + IFN-α condition and percentages are shown as mean ± SEM (n=3). Stimulation conditions were compared with medium condition for each input population using paired, two-sided t-tests with Holm-Šídák correction for multiple testing (adjusted p-values: <.05(*), <.005(**)). (k) Two potential models explaining pDC and cDC potential in lo-lo cells.
