# Supplementary material for: Ly6D+Siglec-H+ precursors contribute to conventional dendritic cells via a Zbtb46+Ly6D+ intermediary stage
Source: Nat Commun. 2022 Jun 16;13:3456. doi: 10.1038/s41467-022-31054-4 (PMC9200809; doi:10.1038/s41467-022-31054-4)
Supplement: Supplementary file 3 — Description of Additional Supplementary Files [file 41467_2022_31054_MOESM3_ESM.pdf]

Supplementary Data 1: Enriched KEGG pathways

This table contains the enriched KEGG pathways for each DEG cluster, as determined by GeneOverlap. For each significant pathway a p-value (Fisher's exact test, Benjamini-Hochberg adjusted) and a list of overlapping genes are reported.

Supplementary Data 2: Enriched TF motifs

This table contains enriched TF motifs for each cluster, determined by RcisTarget. The 50 most significant hits are reported. NES, normalized enrichment score; AUC, area under curve; TF_direct, inferred TF binding to the specified motif; nEnrGenes, number of enriched genes; rankAtMax, ranking at the maximum enrichment, used to determine the number of enriched genes; enrichedGenes, list of enriched genes.
