# Supplementary material for: Ly6D+Siglec-H+ precursors contribute to conventional dendritic cells via a Zbtb46+Ly6D+ intermediary stage
Source: Nat Commun. 2022 Jun 16;13:3456. doi: 10.1038/s41467-022-31054-4 (PMC9200809; doi:10.1038/s41467-022-31054-4)
Supplement: Supplementary file 6 — Reporting Summary [file 41467_2022_31054_MOESM6_ESM.pdf]

nature research

Corresponding author(s): Anne Krug

Last updated by author(s): May 24, 2022

Reporting Summary

Nature Research wishes to improve the reproducibility of the work that we publish. This form provides structure for consistency and transparency in reporting. For further information on Nature Research policies, see our Editorial Policies and the Editorial Policy Checklist.

Statistics

For all statistical analyses, confirm that the following items are present in the figure legend, table legend, main text, or Methods section.

n/a	Confirmed	
☐	☒	The exact sample size (*n*) for each experimental group/condition, given as a discrete number and unit of measurement
☐	☒	A statement on whether measurements were taken from distinct samples or whether the same sample was measured repeatedly
☐	☒	The statistical test(s) used AND whether they are one- or two-sided<br>*Only common tests should be described solely by name; describe more complex techniques in the Methods section.*
☒	☐	A description of all covariates tested
☐	☒	A description of any assumptions or corrections, such as tests of normality and adjustment for multiple comparisons
☐	☒	A full description of the statistical parameters including central tendency (e.g. means) or other basic estimates (e.g. regression coefficient) AND variation (e.g. standard deviation) or associated estimates of uncertainty (e.g. confidence intervals)
☐	☒	For null hypothesis testing, the test statistic (e.g. *F*, *t*, *r*) with confidence intervals, effect sizes, degrees of freedom and *P* value noted<br>*Give P values as exact values whenever suitable.*
☒	☐	For Bayesian analysis, information on the choice of priors and Markov chain Monte Carlo settings
☒	☐	For hierarchical and complex designs, identification of the appropriate level for tests and full reporting of outcomes
☒	☐	Estimates of effect sizes (e.g. Cohen's *d*, Pearson's *r*), indicating how they were calculated

Our web collection on statistics for biologists contains articles on many of the points above.

Software and code

Policy information about availability of computer code

Data collection	Flow cytometry data was collected using either SpectroFlo v2.2 (Cytek Aurora), CytExpert v2.1 (CytoFlex) or FACSDiva v9.0 (Fortessa, Aria Fusion), qPCR data was collected using LightCycler 480 Software v1.5
Data analysis	FlowJo 10.7.1 was used for analysis of Flow Cytometry data. FlowJo plugin UMAP v3.1 was further used.<br>Microsoft Excel 16.0.12827.20236 64 bit was used for structuring data and working on tabular data.<br>Python 3.7.6 64bit was used for data preparation and RNA velocity analysis (important packages: scvelo v0.2.1, velocyto v0.17,)<br>R version 3.5 was used for bulk RNA-seq analysis, v3.6.2 was used for all single-cell RNA-seq analysis.<br>R packages:<br>zUMIs v0.0.1 (bulk RNA) 2.5.6 (single-cell RNA), STAR v2.5.2b (bulk RNA), v2.6.0 (single-cell RNA), DESeq2 v1.18.1, RcisTarget v1.0.2, GeneOverlap v1.14.0, MSigDB v6.1<br>All bioinformatical analysis was done using previously published R or python packages. For analysis of our data we followed the best practices found in the respective vignettes for each package, i.e. no custom algorithms were used.

For manuscripts utilizing custom algorithms or software that are central to the research but not yet described in published literature, software must be made available to editors and reviewers. We strongly encourage code deposition in a community repository (e.g. GitHub). See the Nature Research guidelines for submitting code & software for further information.

Data

Policy information about availability of data

All manuscripts must include a data availability statement. This statement should provide the following information, where applicable:

- Accession codes, unique identifiers, or web links for publicly available datasets
- A list of figures that have associated raw data
- A description of any restrictions on data availability

Source data supporting the findings of this study are provided in the specified file with this paper. Bulk RNA-seq data that support the findings of this study have been deposited in the Gene Expression Omnibus (GEO) NCBI database under Accession No. GSE189780. Single-cell RNA-seq data that support the findings of this study have been deposited at the European Nucleotide Archive under Accession No PRJEB52646 and ArrayExpress (https://www.ebi.ac.uk/arrayexpress/experiments/E-MTAB-11752/) and in processed form for simplified downstream analysis with scvelo at figshare under https://figshare.com/articles/dataset/pDC_precursor_scvelo_h5ad/17013788/1 (DOI: 10.6084/m9.figshare.17013788.v1). The mouse reference genome mm10 (GRCm38 release 84) used in this study can be found under GeneBank Accession No.: GCA_000001635.2 (https://www.ncbi.nlm.nih.gov/assembly/GCF_000001635.20/). Molecular Signature Database gene sets are available via the respective R package MSigDB. Further data acquired and analysed in this study are available from the corresponding author upon reasonable request.

Field-specific reporting

Please select the one below that is the best fit for your research. If you are not sure, read the appropriate sections before making your selection.

- [x] Life sciences
- [ ] Behavioural & social sciences
- [ ] Ecological, evolutionary & environmental sciences

For a reference copy of the document with all sections, see nature.com/documents/nr-reporting-summary-flat.pdf

Life sciences study design

All studies must disclose on these points even when the disclosure is negative.

Sample size	Sample size calculation was not performed in advance for this exploratory study. Sample size was chosen based on experience from prior similar experiments and data variability.
Data exclusions	Exclusion criteria were not pre-established. Samples were excluded if technical problems arose during the experiment (e.g. faulty equipment or errors in the experimental process). For single cell RNA sequencing the filtering criteria are described in detail in the methods section.
Replication	The number of replicates is indicated in each figure legend. All attempts for replication were successful. For sorted cell populations, multiple (2-10) sex- and age-matched mice were pooled to acquire enough cells.
Randomization	Age- and/or sex-matched littermates were used for experiments whenever possible. Mice were cohoused in the same rack and room. For comparison of different cell populations within an experiment, these were sorted at the same time from the same bone marrow cell sample.
Blinding	Investigators were not blinded during data acquisition and data analysis, because the same data acquisition settings and data analysis settings (compensation and gating) were applied to all samples of an experiment in flow cytometric data analysis precluding a subjective influence of knowledge of the sample name and belonging to sample groups on the data.<br>Genotyping of mice was conducted by an independent person.

Reporting for specific materials, systems and methods

We require information from authors about some types of materials, experimental systems and methods used in many studies. Here, indicate whether each material, system or method listed is relevant to your study. If you are not sure if a list item applies to your research, read the appropriate section before selecting a response.

Materials & experimental systems

n/a	Involved in the study	
[ ]	[x]	Antibodies
[ ]	[x]	Eukaryotic cell lines
[x]	[ ]	Palaeontology and archaeology
[ ]	[x]	Animals and other organisms
[x]	[ ]	Human research participants
[x]	[ ]	Clinical data
[x]	[ ]	Dual use research of concern

Methods

n/a	Involved in the study	
[x]	[ ]	ChIP-seq
[ ]	[x]	Flow cytometry
[x]	[ ]	MRI-based neuroimaging

Antibodies

Antibodies used

Target | Fluorophore | Clone | Dilution | Manufacturer | Catalog Nr
B220 | BV605 | RA3-6B2 | 1:200 | BioLegend | 103243
B220 | FITC | RA2-6B2 | 1:200 | eBioscience | 14-0452-82
B220 | BV605 | RA3-6B2 | 1:200 | BD | 563708
BST-2 | APC | eBio129c | 1:200 | eBioscience | 17-3171-82
CCR4 | PE | 2G12 | 1:200 | BioLegend | 131204
CCR5 | BUV737 | C34-3448 | 1:100 | BD | 749670
CCR5 | PE | HM-CCR5 | 1:200 | BioLegend | 107005
CCR9 | PE | eBioCW-1.2 | 1:200 | eBioscience | 12-1999-42
CCR9 | eF450 | CW-1.2 | 1:100 | eBioscience | 48-1991-82
CD115 | PE-Cy7 | AFS98 | 1:200 | BioLegend | 135524
CD115 | BUV737 | AFS98 | 1:200 | BD | 750948
CD115 | APC | AFS98 | 1:200 | BioLegend | 135510
CD117 | BV510 | ACK2 | 1:200 | BioLegend | 105839
CD117 | PE | 2B8 | 1:200 | BioLegend | 161605
CD11b | APC | M1/70 | 1:200 | BioLegend | 101212
CD11b | PerCP-Cy5,5 | M1/70 | 1:200 | BioLegend | 101228
CD11b | BV570 | M1/70 | 1:200 | BioLegend | 101233
CD11c | PE-Cy7 | N418 | 1:200 | BioLegend | 117318
CD11c | PE-Cy7 | N418 | 1:200 | eBioscience | 25-0114-82
CD11c | APC-Cy7 | HL3 | 1:200 | BD | 561241
CD11c | BUV395 | HL3 | 1:200 | BD | 564080
CD135/Flt3 | APC | A2F10 | 1:100 | BioLegend | 135310
CD135/Flt3 | BV421 | A2F10 | 1:100 | BioLegend | 135313
CD14 | PE | Sa2-8 | 1:200 | BioLegend | 123310
CD16/32 | PerCP-Cy5,5 | 93 | 1:200 | BioLegend | 101324
CD16/32 | BUV496 | 93 | 1:200 | BD | 751694
CD19 | APC-Cy7 | 6D5 | 1:200 | BioLegend | 115530
CD19 | FITC | 1D3 | 1:200 | BD | 553785
CD2 | BUV615 | RM2-5 | 1:100 | BD | 751141
CD24 | APC | M1/69 | 1:200 | BioLegend | 101814
CD24 | BV605 | M1/69 | 1:200 | BioLegend | 101827
CD3 | APC-Cy7 | 145-2C11 | 1:200 | BioLegend | 100222
CD3 | FITC | 145-2C11 | 1:200 | BD | 553061
CD4 | APC | RM4-5 | 1:200 | BioLegend | 100412
CD4 | BUV661 | SK3 | 1:200 | BD | 612974
CD45.1 | PE-Cy7 | A20 | 1:200 | eBioscience | 110730
CD45.1 | AF700 | A20 | 1:200 | BioLegend | 110724
CD45.2 | APC | 104 | 1:200 | BioLegend | 109814
CD45.2 | Alexa Flour 700 | 104 | 1:200 | BioLegend | 109822
CD64 | APC | X54-5/7.1 | 1:200 | BioLegend | 139306
CD81 | Alexa 350 | 431301 | 1:200 | Novus Bio | NB100-65805AF350
CD86 | BV650 | GL-1 | 1:200 | BioLegend | 105036
CD8a | PerCP | 53-6.7 | 1:200 | eBioscience | MCD0831
CD8a | PE | 53-6.7 | 1:200 | BD | 553033
CD9 | APC | eBioKMC8 | 1:200 | eBioscience | 17-0091-82
CX3CR1 | BV711 | SA011F11 | 1:200 | BioLegend | 149031
CX3CR1 | PE | SA011F11 | 1:200 | BioLegend | 149006
CXCR3 | PerCP | CXCR3-173 | 1:100 | eBioscience | 45-1831-82
CXCR4 | APC | 2B11 | 1:200 | eBioscience | 17-9991-82
CXCR5 | APC | L138D7 | 1:200 | BioLegend | 145506
F480 | BUV805 | T45-2342 | 1:400 | BD | 749282
IL7R/CD127 | PerCP-Cy5.5 | A7R34 | 1:200 | BioLegend | 135022
IL7R/CD127 | PE-Cy7 | A7R34 | 1:200 | BioLegend | 135014
Ly49Q | FITC | 2E6 | 1:200 | MBL/Novus | d160-4
Ly6C | PE-Dazzle594 | HK1.4 | 1:400 | BioLegend | 128044
Ly6C | AF700 | HK1.4 | 1:200 | BioLegend | 128024
Ly6D | PE | 49-H4 | 1:800 | BioLegend | 138604
Ly6G | APC-Cy7 | 1A8 | 1:200 | BioLegend | 127624
Ly6G | FITC | 1A8 | 1:200 | BioLegend | 127606
MHCII | APC-eFluor780 | M5/114.15.2 | 1:400 | eBioscience | 47-5321-82
MHCII | BV650 | M5/114.15.2 | 1:400 | BioLegend | 107641
MHCII | BV650 | M5/114.15.2 | 1:400 | eBioscience | 64-5321-82
MHCII | Spark Blue 550 | M5/114.15.2 | 1:400 | BioLegend | 107662
NK1.1 | APC-Cy7 | PK136 | 1:200 | BioLegend | 108724

NK1.1 | FITC | PK136 | 1:200 | BD | 553164
Sca1 | APC | D7 | 1:200 | BioLegend | 108112
Siglec-H | BV786 | 440c | 1:200 | BD | 752580
Siglec-H | AF488 | 440c | 1:200 | BioLegend | 129611
Siglec-H | FITC | 440c | 1:200 | BioLegend | 129604
Siglec-H | AF647 | 440c | 1:200 | BioLegend | 129608
Sirp-alpha | PerCP-Cy5.5 | P84 | 1:200 | BioLegend | 144010
Sirp-alpha | Alexa Flour 700 | P84 | 1:200 | BioLegend | 144022
Ter119 | APC-Cy7 | TER-119 | 1:200 | BioLegend | 116223
Ter119 | FITC | TER-119 | 1:200 | BioLegend | 116206
XCR1 | Alexa Flour 647 | ZET | 1:200 | BioLegend | 148214

Dyes:
fixable viability dye eF506 | 1:1000 | ThermoFisher | Cat No. 65-0866-18
CellTrace Blue proliferation dye | 5 µM| ThermoFisher | C34574
Zombie Red fixable viability dye | 1:1000 | BioLegend | 423109
Zombie NIR fixable viablity dye | 1: 1000 | BioLegend | 423105

Validation

Commercially available antibodies were validated by the respective manufacturers for species reactivity and application in flow cytometry (see informations from the companies' websites below). All antibodies were further tested by flow cytometry in our lab to define the optimal dilution factor (by serial dilution).

BioLegend: "Specificity testing of 1-3 target cell types with either single- or multi-color analysis (including positive and negative cell types). Once specificity is confirmed, each new lot must perform with similar intensity to the in-date reference lot. Brightness (MFI) is evaluated from both positive and negative populations. Each lot product is validated by QC testing with a series of titration dilutions."

BD Biosciences: "We conduct quality control (QC) testing in primary model systems to ensure biological accuracy in an ISO 9001 certified facility. BD carefully selects and characterizes antibody content in product development and tests in relevant primary model systems to ensure biological accuracy. BD conducts rigorous QC testing of each antibody lot tested side-by-side with a previously produced lot as reference. Our product development process includes testing on a combination of primary cells, cell lines and/or transfectant cell models with relevant controls using multiple immunoassays to ensure biological accuracy. We also perform multiplexing with additional antibodies to interrogate antibody staining in multiple cell populations. BD believes antibody validation is critical to ensure accurate scientific results. Both the consumer and the reagent provider share the responsibility for reproducible science."

eBioscience/Invitrogen: "Invitrogen antibodies are currently undergoing a rigorous two-part testing approach. Part 1—Target specificity verification: This helps ensure the antibody will bind to the correct target. Our antibodies are being tested using at least one of the following methods to ensure proper functionality in researcher's experiments. Part 2—Functional application validation. These tests help ensure the antibody works in a particular application(s) of interest, which may include Flow Cytometry. Most antibodies were developed with specific applications in mind. Testing that an antibody generates acceptable results in a specific application is the second part of confirming antibody performance.

Novus Biologicals: "Genetic Strategy Validation- Expression of the target protein is compared before and after knockout or knockdown using CRISPR/CAS9 or siRNA/shRNA. If protein expression following knockout or knockdown is substantially reduced, then antibody specificity is ensured. Orthogonal Validation- The target protein is examined with an antibody independent strategy and compared with results from an antibody-dependent strategy. A correlation between these two strategies indicates specificity between the antibody and its target protein. Examples of antibody independent techniques may include in situ hybridization, quantitative PCR, RNA-seq or mass spectrometry. Independent Antibody Validation- The data generated using several antibodies (ideally targeting different epitopes) in the same protein is compared (e.g. molecular weight and cellular localization). Consistent results imply antibody selectivity to the target protein. Expression of Tagged Proteins Validation- A tagged protein is used as a standard for comparison in Western blotting and/or immunocytochemistry (ICC). For example, if the distribution of the tagged protein overlaps with the immunofluorescence signal, then antibody specificity is confirmed. Biological Strategies Validation- These strategies use defined biological or chemical modulation of protein expression to demonstrate antibody specificity to the target protein. The data is compared across multiple cell lines including positive and negative expressing cells, and multiple species, if applicable".

Eukaryotic cell lines

Policy information about cell lines

Cell line source(s)

EL08-1D2 stromal cell line: Robert AJ Oostendorp, Technical University of Munich, School of Medicine, Department of Internal Medicine III, Ismaninger Str. 22, 81675 Munich, Germany
(Oostendorp RA, et al. Stromal cell lines from mouse aorta-gonads-mesonephros subregions are potent supporters of hematopoietic stem cell activity. Blood 99, 1183-1189 (2002)).

CHO-flk2 (Flt3L-producing) cell line: Nic Nicola, Walter and Eliza Hall institute, Melbourne, Australia (Rasko JE et al. The flt3/flk-2 ligand: receptor distribution and action on murine haemopoietic cell survival and proliferation. Leukemia. 1995 Dec;9 (12):2058-66.
Format:)

Authentication

Cell morphology was monitored regularly. Cell lines were kept in culture for short time periods by design. CHO-flk2 cell supernatants were checked for Flt3L concentration by ELISA and for bioactivity in Flt3L BM cell cultures. Other authentication

procedures were not used.

Mycoplasma contamination	Cell lines were routinely tested for mycoplasm contamination by PCR and found to be negative.
Commonly misidentified lines (See ICLAC register)	Not used

Animals and other organisms

Policy information about studies involving animals; ARRIVE guidelines recommended for reporting animal research

Laboratory animals	Adult mice of both sexes (2 to 8 months old) were used for isolation of cells for ex vivo analysis and in vitro experiments. All mice had C57BL/6 background.<br>Mice were maintained in specific pathogen-free SPF conditions with a 12 hour dark/light cycle, in individually vented cages (IVCs, type II long cages, measuring 18x30x13 cm with stocking density according to EU guideline 2010/63) supplied with autoclaved bedding, play tunnels, nestles and mouse houses. Irradiated food and sterile filtered and UV-light exposed water were provided ad libitum. Cage manipulations took place in laminar flow hoods. Air temperature was 22 ± 2°C and humidity 55 ± 10% with daily control and record.
Wild animals	The study did not involve wild animals
Field-collected samples	The study did not involve field-collected samples.
Ethics oversight	All experimental procedures involving mice were performed in accordance with the regulations of, and approved by, the local government (Regierung von Oberbayern).

Note that full information on the approval of the study protocol must also be provided in the manuscript.

Flow Cytometry

Plots

Confirm that:

- [x] The axis labels state the marker and fluorochrome used (e.g. CD4-FITC).
- [x] The axis scales are clearly visible. Include numbers along axes only for bottom left plot of group (a 'group' is an analysis of identical markers).
- [x] All plots are contour plots with outliers or pseudocolor plots.
- [x] A numerical value for number of cells or percentage (with statistics) is provided.

Methodology

Sample preparation	Bone marrow cells were isolated from femura, tibiae and hip bones by flushing with RPMI 1640 and passed through a 100 µm strainer. Spleens were cut into pieces and digested for 30 minutes at 37°C in DC medium containing DNAse I (Sigma) and Collagenase D (Sigma), then passed through 100 µm and 40µm cell strainers. After red blood cell lysis using Red Blood Cell Lysing Buffer (Sigma) for 5 minutes at RT and washing with RPMI 1640 BM and spleen cells were resuspended in FACS buffer (PBS, 2% FCS, 2 mM EDTA).
Instrument	Cytek Aurora, BD LSRFortessa, BD FACS Aria III, BD FACSAria Fusion, CytoFLEX S (Beckman Coulter), LightCycler 480
Software	SpectroFlo v2.2 (Cytek Aurora), CytExpert v2.1 (CytoFlex), FACSDiva v9.0 (Aria Fusion), LightCycler™ 480 Software v1.5
Cell population abundance	Fractions of the sorted populations were re-analyzed after the sort and the purity was generally > 90%, with divergences usually stemming from shifts in fluorescence due to the duration of the sort paired with strict sort gates, or some cells dying after sorting.
Gating strategy	Gating strategies are shown in the supplmentary material. In general all samples were initially gated on FSC-A vs SSC-A plot, thendoublets were excluded using the FSC-A vs FSC-H plot and life cells were gated as cells negative for fixable viability dye eFluro506 or Zombie-NIR in the FSC-A vs live/dead stain plot.<br>Steady-state analysis of Zbtb46-eGFP BM and spleen:<br>CDP: Lin-/CD11c+/MHCII-/CD135+/CD115+/CD117int<br>CLP: Lin-/B220-/Ly6C-/CD135+/CD117lo-int/CD115-/IL7R+<br>B cells: Lin+/B220+/CD11b-/Ly6D+/Zbtb46-/CCR9-<br>Macrophages: Large cells/Lin+/F480+/MHCII+<br>CD4 T cells: small cells/Lin+/Ly6D-/Zbtb46-/MHCII-/CD11c-/CD4+<br>CD8 T cells small cells/Lin+/Ly6D-/Zbtb46-/MHCII-/CD11c-/CD8a+<br>MDP: Lin-/CD11c+/MHCII-/CD135+/CD115+/CD117high<br>cDC1: Lin-/CD11c+/MHCII+/Zbtb46+/Ly6D-/XCR1+/Sirp-alpha-/CD8a+<br>cDC2: Lin-/CD11c+/MHCII+/Zbtb46+/Ly6D-/XCR1-/CD11b+/Sirp-alpha+<br>pre-cDC1: Lin-/CD11c+/B220-/Siglec-H-/Ly6D-/MHCIIint/Zbtb46+/Ly6C-<br>pre-cDC2: Lin-/CD11c+/B220-/Siglec-H-/Ly6D-/MHCIIint/Zbtb46+/Ly6C+<br>Siglec-H+ pre-cDC: Lin-/CD11c+/Siglec-H+/Ly6D-/Sirp-alpha-/MHCII-/CD135+/Zbtb46+

Siglec-H- pre-cDC: Lin-/CD11c+/Siglec-H-/B220-/Sirp-alpha-/MHCII-/CD135+/Zbtb46+
Ly6D+ LP: Lin-/B220-/Ly6C-/CD135+/CD117lo-int/CD115-/IL7R+/Ly6D+/
Ly6D+ Siglec-H+ LP: Lin-/B220-/Ly6C-/CD135+/CD117lo-int/CD115-/IL7R+/Ly6D+/Siglec-H
pre-pDC: Lin-/CD11c-/MHCII-/CD115+/CD117int/Ly6D+/SigH+/CD115-/CD2int/IL7R+/CD81+
lo-lo: Lin-/CD11c+/Siglec-H+/Ly6D+/B220low/CCR9low
lo-hi: Lin-/CD11c+/Siglec-H+/Ly6D+/B220high/CCR9low
pDC: Lin-/CD11c+/Siglec-H+/Ly6D+/B220high/CCR9high
Zbtb46+ Ly6D+: Lin-/CD11c+/Siglec-H+/Ly6Dint-high/Zbtb46+

☒ Tick this box to confirm that a figure exemplifying the gating strategy is provided in the Supplementary Information.
